# Bibliometric analysis of publications on necroptosis from 2001 to 2021

**DOI:** 10.3389/fcell.2022.946363

**Published:** 2022-09-20

**Authors:** Yang Liu, Xiaojiang Zhou, Fangfei Wang, Cong Liu, Jun Xie, Le Guan, Yong Xie

**Affiliations:** ^1^ Department of Gastroenterology, The First Affiliated Hospital of Nanchang University, Nanchang, Jiangxi, China; ^2^ Jiangxi Clinical Research Center for Gastroenterology, Nanchang, Jiangxi, China; ^3^ School of Basic Medical Sciences, Fujian Medical University, Fuzhou, Fujian, China

**Keywords:** necroptosis, bibliometric analysis, hotspot, trend, cancer

## Abstract

**Background:** Necroptosis plays an important role in inflammation, cancer, and neurodegenerative diseases. In recent years, the number of studies related to necroptosis has increased and research has become increasingly in-depth. This study aimed to summarize the research conducted since 2001 to discover hotspots and trends in the field of necroptosis.

**Methods:** The Web of Science Core database was used to identify global publications on necroptosis from 2001 to 2021. Bibliometric analysis was performed using Rstudio, VOSviewer, and CiteSpace.

**Results:** The number of publications related to necroptosis gradually increased from 2001 to 2021. Vandenabeele P had the most publications at 45. Yuan JY had the most citations at 5,901. Necroptosis research has been dominated by China and Chinese institutions. Cell Death and Disease had the highest number of related publications among the examined journals. Seven of the top 10 most cited papers had more than 500 citations. Necroptosis, cell death, autophagy, injury, cancer, activated B cell nuclear factor kappa-light chain enhancer, and oxidative stress were important keywords in keyword analysis. Recent research has increasingly focused on breast cancer, receptor-interacting serine/threonine protein kinase 1, modulation, pseudokinase mixed lineage kinase domain-like protein, membrane, protection, and cycle.

**Conclusion:** Interest in necroptosis-related research continues to increase steadily, and there is close cooperation between countries and institutions in the field of necroptosis. The study of necroptosis-related molecules and mechanisms, and the relationship between necroptosis and cancer, may be hotspots and directions in future research.

## 1 Introduction

Necroptosis is a form of regulated necrotic cell death mediated by receptor-interacting serine/threonine protein kinase (RIPK) one and RIPK3. Necroptosis is characterized by early loss of integrity of the plasma membrane, intracellular contents leakage, and organelle swelling. Cells that die through necroptosis lack typical apoptotic characteristics. Degterev et al. (2005) reported that the small-molecule inhibitor necrostatin-1 (Nec-1) hinders necrotic cell death. In the absence of caspase-8, necroptosis is a form of regulated necrotic cell death (Newton et al., 2019a; Newton et al., 2019b) distinct from apoptosis (Wang et al., 2018).

Necroptosis signaling pathways include both canonical and noncanonical pathways. The most representative canonical signaling pathway is mediated by extracellular tumor necrosis factor (TNF) and RIP1-dependent kinase activity. Ligand-dependent death receptors, including Fas cell surface death receptor (Fas), TNFR1, and TNF-related apoptosis-inducing ligand (TRAIL) receptors, can initiate the necroptosis pathway (Choi et al., 2019). TNF stimulation of TNFR1 has three functional outcomes. Depending on the assembly of different regulatory proteins, the different pathways ultimately lead to inflammation induced by nuclear factor kappa-light-chain-enhancer of activated B cells (NF-κB), caspase-8-mediated apoptosis, or the activation of alternative necroptosis pathways when caspase-8 is inhibited (Choi et al., 2019). The non-canonical pathway refers to necroptosis initiated upon stimulation by other factors independent of RIPK1. In addition to RIPK1, other RIP homotypic interaction motif (RHIM)-containing molecules, including TIR domain-containing adaptor-inducing interferon-β (TRIF) and DNA-dependent activator of interferon-regulatory factors (DAI), which can also interact with RIPK3 through their domains to initiate downstream signals that ultimately lead to necroptosis.

Pathogens can induce the formation of necrosome in a manner dependent on RHIM of RIPK3 (Huang et al., 2018; Zhang et al., 2020). RIP1 and RIP3 are key proteins involved in the formation of the necrosome complex during TNF-mediated necroptosis (Zhao et al., 2012). After activation of RIPK3, it activates the pseudokinase mixed lineage kinase domain-like protein (MLKL) through phosphorylation, which plays a critical role in the induction of necrosis. MLKL acts in two ways: as a platform for the recruitment of sodium or calcium ion channels into the plasma membrane (Xia et al., 2016) and through the formation of pores in the plasma membrane promoted by the interaction of positively charged amino acids at the amino terminus of MLKL with phospholipids (Xiao et al., 2022).

Necroptosis plays an important role in inflammation, cancer, and neurodegenerative diseases (Beretta and Zaffaroni, 2022; Jayaraman, 2022; Xiao et al., 2022), and research in this field has increased annually. We hope that the scientific output on necroptosis can be reasonably and effectively analyzed from multiple perspectives and aspects to contribute to this field and even to immunology.

Bibliographic analysis is a statistical method used to analyze publications relevant to a specific topic. It is a vital tool for rapidly acquiring useful information and evaluating important research areas and expected future trends. Bibliometric analyses are useful for helping scholars understand new directions and future aspects of research and to obtain quantitative analysis results of parameters related to scientific output in the related field, providing robust support for the design and development of future scientific research (Ma et al., 2020; Shao, 2022). In this study, VOSviewer and CiteSpace were used to analyze the published literature on necroptosis collected from the Web of Science Core Collection. We aimed to present a comprehensive perspective analysis on the field of necroptosis to discover research hotspots and future research directions.

## 2 Methods

### 2.1 Search strategies and data collection

We extracted articles related to necroptosis published in the Web of Science Core Collection (WOSCC) database between 2001 and 2021. We limited the type of document to articles, the language to English, and set the search term as “necroptosis” OR “necroptotic”. The extracted information was downloaded in the corresponding format, and the complete records and references were extracted for analysis.

The selection and extraction of the literature were carried out independently by two researchers (FW and CL) to guarantee the reliability of the results. Among the selected articles, we extracted and analyzed elements that included the number of publications, citations, countries or regions of origin, participating institutions, authors, journals, and keywords. We also searched for the categories of impact factors and journal citation reports for each journal in 2020, which were used to objectively evaluate the quality and value of each journal in the field.

### 2.2 Data analysis

VOSviewer 1.6.16 is widely used for network construction and visualization based on publications, journals, authors, institutes, countries, or keywords (Sastranegara, 2021). We used this software to analyze the co-occurrence of different countries, institutions, authors, and keywords in this study. VOSviewer produces networks that display node sizes relative to the number of publications, where larger nodes represent more publications. The connection between nodes represents the association between different countries, participating institutions, authors, keywords, and references, and the thickness of the connection indicates the strength of the association. Furthermore, CiteSpace 5.8R3c is a useful scientometric tool for analyzing research trends and active fields in the scientific community (Chen et al., 2010). In this study, top-burst keywords in necroptosis research were constructed and visualized using CiteSpace analysis.

We also performed data analyses and visualizations, such as the annual and cumulative number of publications, using Microsoft Excel 2019 and GraphPad Prism 9. We downloaded and installed the Bibliometrix package in Rstudio (Aria and Cuccurullo bibliometrix, 2017) and then imported relevant indicators for data analysis, such as the number of citations, and Lotka’s Law analysis.

## 3 Results

**FIGURE 1:**
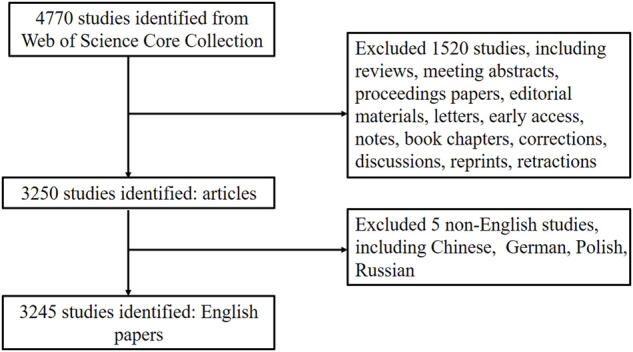
Flowchart of literature selection.

According to the method described above, we retrieved 4,770 articles published since 2001. Among these articles, 4,758 (99.75%) were published in English, five (1.04%) in French, four (0.08%) in German, one (0.02%) in Chinese, one (0.02%) in Russian, one (0.02%) in Japanese, and one (0.02%) in Polish. Through literature screening, we finally included 3,245 English articles for analysis. The specific process is illustrated in Figure 1.

### 3.1 Publication trend

**FIGURE 2:**
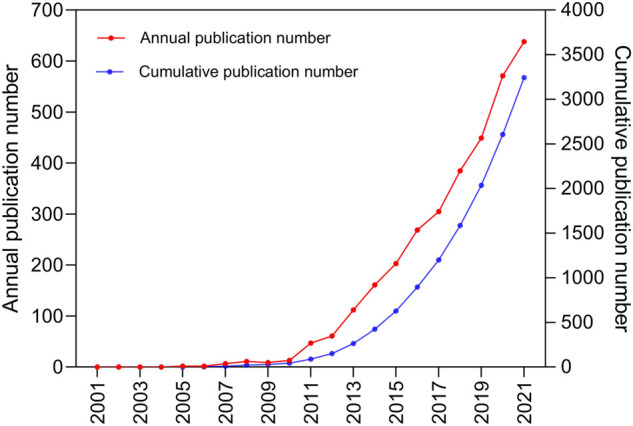
The pattern of the annual and cumulative number of publications in the period from 2001 to 2021.

Annual and cumulative publication volumes are shown in Figure 2. We found that the number of published articles has steadily increased every year. Between 2001 and 2012, there were less than 100 publications. The annual number of publications increased relatively slowly, from 2005 (two publications) to 2012 (61 publications). The annual number of articles has increased rapidly, from 2013 (112 publications) to 2021 (638 publications). Between 2013 and 2021, 3,039 necroptosis-related publications were published, accounting for 95.3% of all publications in the past 20 years.

### 3.2 Analysis of the author and co-author analysis

**FIGURE 3:**
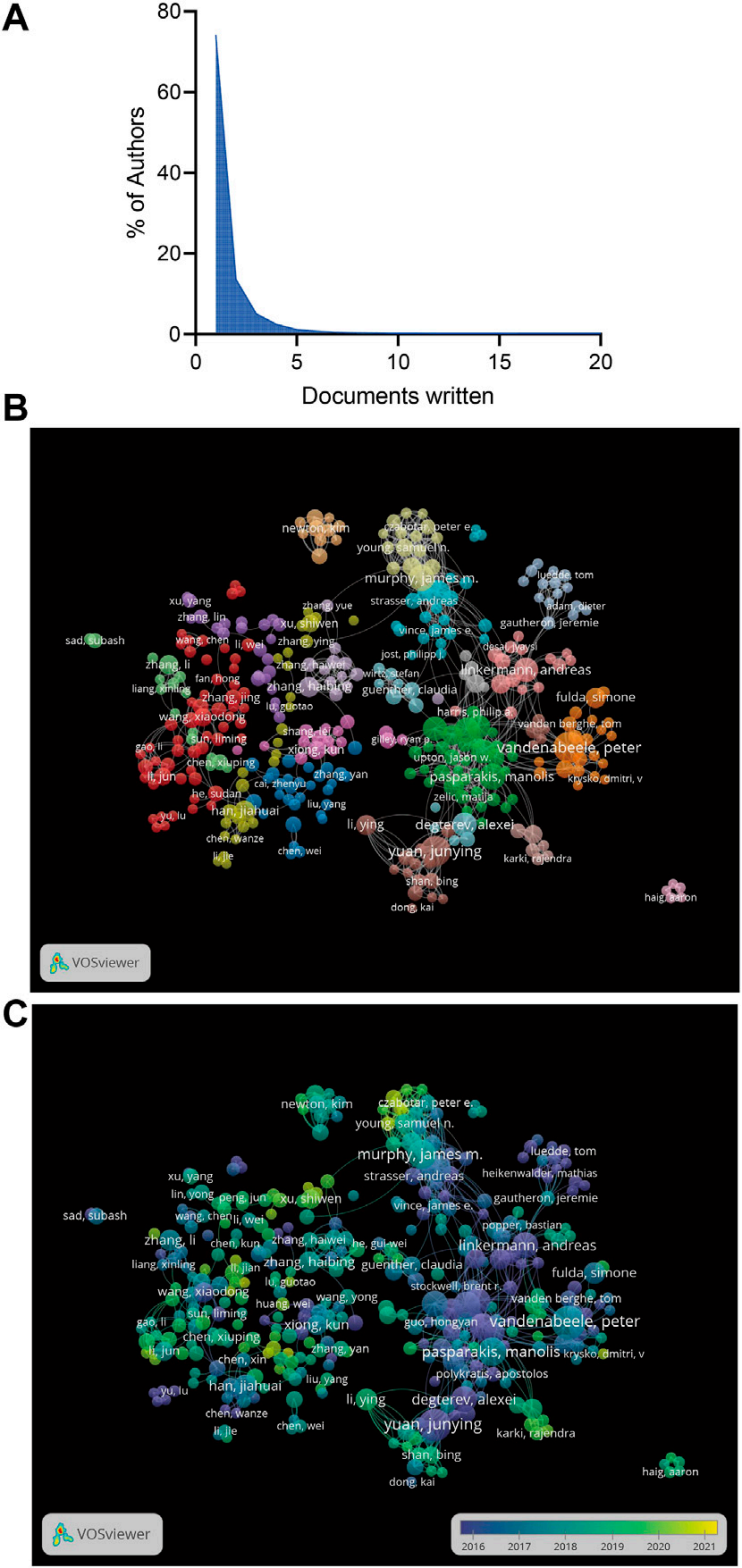
**(A)**The frequency distribution of scientific productivity (Lotka’s Law). **(B)** The network map of authors for necroptosis research. **(C)** The overlay visualization of authors for necroptosis research.

The 3,245 articles had 19671 authors. The top 10 authors by publication volume are summarized in Table 1. According to the number of publications, Vandenabeele P (45, 0.014%) ranked first, followed by Yuan JY (43, 0.013%), Murphy JM (33, 0.010%), Pasparakis M (33, 0.010%), and Silke J (29, 0.009%). Lotka law describes the relationship between an author and the number of papers they have written, and 74.20% of the authors contributed only one publication (Figure 3A). In the co-authorship analysis, we included 500 authors with at least five publications, and the largest cluster in the author relationship network was red, with 56 authors (Figure 3B).

**TABLE 1:** The top 10 productive authors and the most-cited authors in the field of necroptosis.

Author	Articles (n)	Author	Total citation (n)
Vandenabeele Peter	45	Yuan JunYing	5,901
Yuan JunYing	43	Degterev Alexei	5,791
James M. Murphy	33	Bertin John	4,883
Pasparakis Manolis	33	Vandenabeele Peter	4,841
Silke John	29	Douglas R. Green	4,776
Bertin John	29	Peter J. Gough	4,634
Douglas R. Green	29	Linkermann Andreas	4,394
Linkermann Andreas	28	Edward S. Mocarski	4,035
Edward S. Mocarski	28	William J. Kaiser	4,014
Degterev Alexei	28	Gregory D. Cuny	3,745

According to the overlay visualization of the authors for necroptosis research, the size of the circle represents the number of articles, and different colors correspond to different years. Wang YY, Zhang J, Xu SW, Young SN and Czabator PE recently produced more articles (Figure 3C). In the initial stage of necroptosis research, Vandenabeele P and Yuan JY contributed more publications, suggesting that the author could be a leading expert in the field. By analyzing the citations of the authors, we observed that Yuan JY had the highest citation (5,901 citations), followed by Degterev A (5,791 citations), Bertin J (4,883 citations), Vandenabeele P (4,841 citations), and Green DR (4,776 citations; Table 1).

### 3.3 Analysis of participating institutions and countries of origin

**FIGURE 4:**
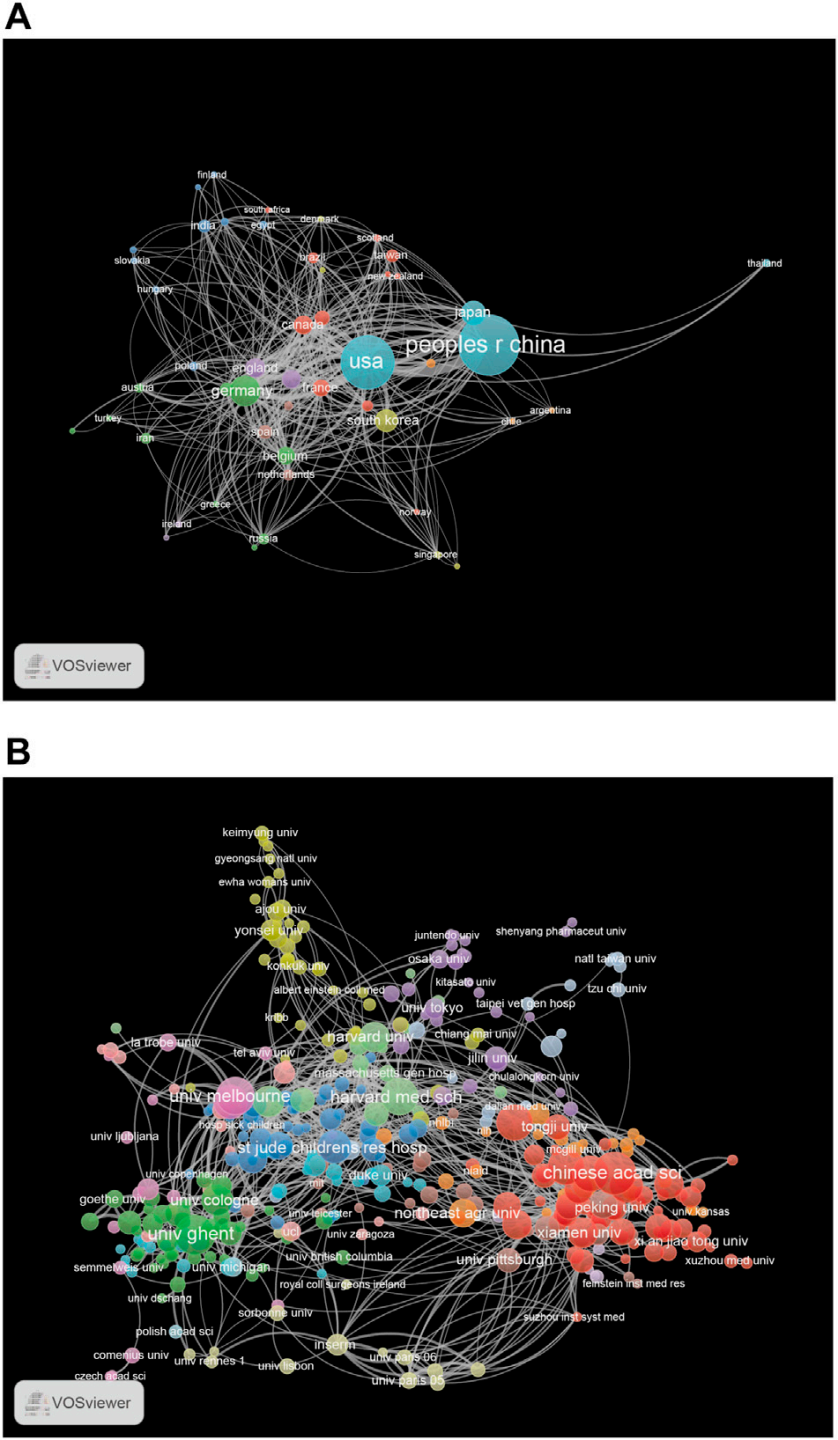
**(A)** The network map of countries for necroptosis research. **(B)** The network map of institutions for necroptosis research.

Research on necroptosis has been conducted by 3,032 institutions in 78 countries. The top 10 countries contributed 2,766 (85.24%) publications, and the top five were China (1,213), the United States (686), Germany (232), Korea (156), and Japan (147; Table 2). From the analysis of the coauthorship of the countries, we found that China, the United States, and Germany were the top three productive countries in this field (Figure 4A).

**TABLE 2:** The top 10 countries and institutions that have contributed to publications on necroptosis research.

Country	Articles (n)	Institution	Articles (n)
China	1,213	UNIV GHENT	143
United States	686	UNIV COLOGNE	121
Germany	232	ZHEJIANG UNIV	119
Korea	156	FUDAN UNIV	114
Japan	147	GENENTECH INC.	113
Australia	86	SHANGHAI JIAO TONG UNIV	110
Canada	79	SOOCHOW UNIV	107
Belgium	63	UNIV MELBOURNE	104
United Kingdom	58	SUN YAT SEN UNIV	102
France	46	HARVARD MED SCH	98

The top 10 institutions published 1,131 articles (35%). Five of them were from China, except for The University of Ghent (Belgium), The University of Cologne (Germany), Genentech Inc. (United States), The University of Melbourne (Australia), and The Harvard Medical School (United States; Table 2). The University of Ghent contributed the highest number of articles among the top 10 institutions, followed by The University of Cologne (121), Zhejiang University (119), Fudan University (114), and Genentech Inc. (113). Institutional coauthorship and visualization analyses were performed using VOSviewer. We observed that 392 research institutes formed clusters of 15 different colors (Figure 4B), which shows frequent close links between different research institutions, especially for institutions in the same cluster (Figure 4B).

### 3.4 Journal analysis

**FIGURE 5:**
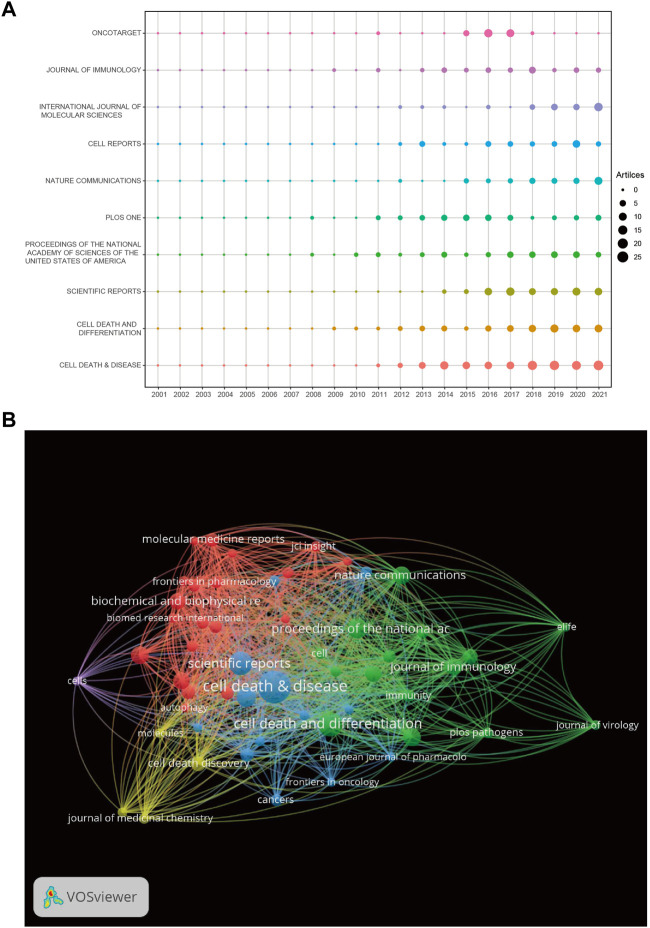
**(A)** The number of publications for the top 10 journals per year. **(B)** The network map of journals for necroptosis research.

A total of 801 journals were found, and the top 10 journals by publication volume in the field of “necroptosis” are summarized in Table 3. In the top 10 journals, 679 (20.92%) articles on necroptosis were published. Cell Death and Disease ranked first, followed by Cell Death and Differentiation, Scientific Reports, Proceedings of the National Academy of Sciences of the United States of America, and PLOS One. The top 10 journals included six from the United States and four from England. Among them, seven journals had impact factors greater than five points, and all journal categories were above Q2, showing that the quality of the articles was excellent. Figure 5A shows the trends in past annual publications for the top 10 journals. Cell Death and Disease, Cell Death and Differentiation, and Scientific Reports have produced many high-quality publications in this field. Cell Reports, Nature Communications, and Oncotarget were relatively new to this field, but developed quickly. The coauthorship and visualization analyses of the journals are shown in Figure 5B. There were five clusters in total, the largest was red (20 items), and there were active collaborations between the journals in the same cluster.

**TABLE 3:** The top 10 journal that have contributed to publications on necroptosis research.

Journal	Articles (n)	Impact factor (2020)	Quartile in category
Cell Death and Disease	166	8.47	Q1
Cell Death Differ	91	15.83	Q1
Scientific Reports	84	4.38	Q1
P NATL ACAD SCI United States	61	11.20	Q1
PLOS One	55	3.24	Q2
Nature Communications	47	14.92	Q1
Cell Reports	46	9.42	Q1
INT J MOL SCI	45	5.92	Q1/Q2
Journal of Immunology	43	5.42	Q2
Oncotarget	41	—	—

Cell Death Differ, Cell Death & Differentiation.

P Natl Acad SCI United States**,** Proceedings of the National Academy of Sciences of the United States of America.

**I**NT J MOL SCI, International Journal of Molecular Sciences.

### 3.5 Cocited reference and keyword cooccurrence cluster analysis

**FIGURE 6:**
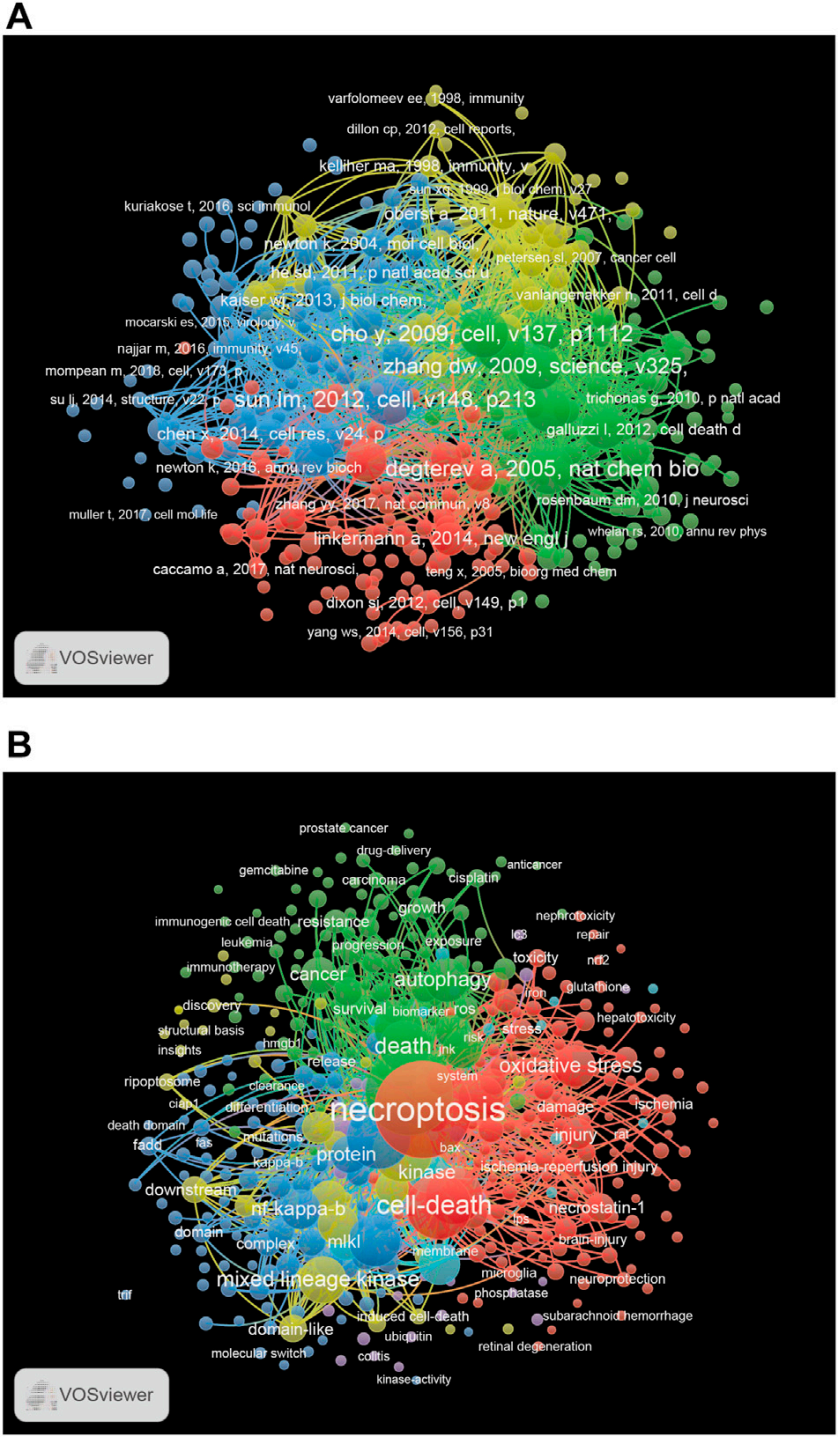
**(A)** The network map of publications for necroptosis research. **(B)** The network map of keywords for necroptosis research.

The 10 most cited papers are listed in Table 4, including four publications with more than 600 citations (Degterev et al., 2005; Cho et al., 2009; He et al., 2009; Sun et al., 2012) and six with 300–600 citations (Degterev et al., 2008; Zhang et al., 2009; Vandenabeele et al., 2010; Cai et al., 2014; Wang et al., 2014; Pasparakis and Vandenabeele, 2015). We identified the most influential references for necroptosis studies using cocitation analysis. Cluster and visualization analyses of the co-cited references were performed. The network map consists of four clusters of different colors (Figure 6A) and a red cluster with the largest number of references, followed by green, blue, and yellow clusters. A total of 9,834 keywords were extracted from the published articles, and a network map was constructed for the keywords that co-occurred more than 10 times (Figure 6B). There were six clusters, including 453 items.

**TABLE 4:** The top 10 most frequently cited references related to necroptosis research.

Type	References	Doi	Citations
Article	Degterev A, 2005, nat chem biol, v1, p112	10.1038/nchembio711	727
Article	Sun lm, 2012, cell, v148, p213	10.1016/j.cell. 2011.11.031	722
Article	Cho Y, 2009, cell, v137, p1112	10.1016/j.cell. 2009.05.037	688
Article	He SD, 2009, cell, v137, p1100	10.1016/j.cell. 2009.05.021	680
Article	Zhang DW, 2009, science, v325, p332	10.1126/science.1172308	572
Article	Degterev A, 2008, nat chem biol, v4, p313	10.1038/nchembio.83	571
Review	Vandenabeele P, 2010, nat rev mol cell bio, v11, p700	10.1038/nrm2970	568
Review	Pasparakis M, 2015, nature, v517, p311	10.1038/nature14191	470
Article	Wang HY, 2014, mol cell, v54, p133	10.1016/j.molcel. 2014.03.003	447
Article	Cai ZY, 2014, nat cell biol, v16, p55	10.1038/ncb2883	354

Article: Research article.

Cluster Red consisted of keywords related to different types of injury (acute kidney injury, brain injury, ischemia-reperfusion injury, and spinal cord injury). Cluster green contained cancer-related keywords, including breast cancer, colorectal cancer, hepatocellular carcinoma, lung cancer, and prostate cancer. Cluster Blue contains immune-related keywords, including caspase-8, cell death, immune response, NOD-like receptor family pyrin domain containing 3 (NLRP3), RIPK1, RIPK3, and TLR. According to the co-occurrence analysis of keywords, we found some important keywords, such as necroptosis, cell death, autophagy, injury, cancer, NF-κB, and oxidative stress.

### 3.6 The analysis of hotspots and frontiers

**FIGURE 7:**
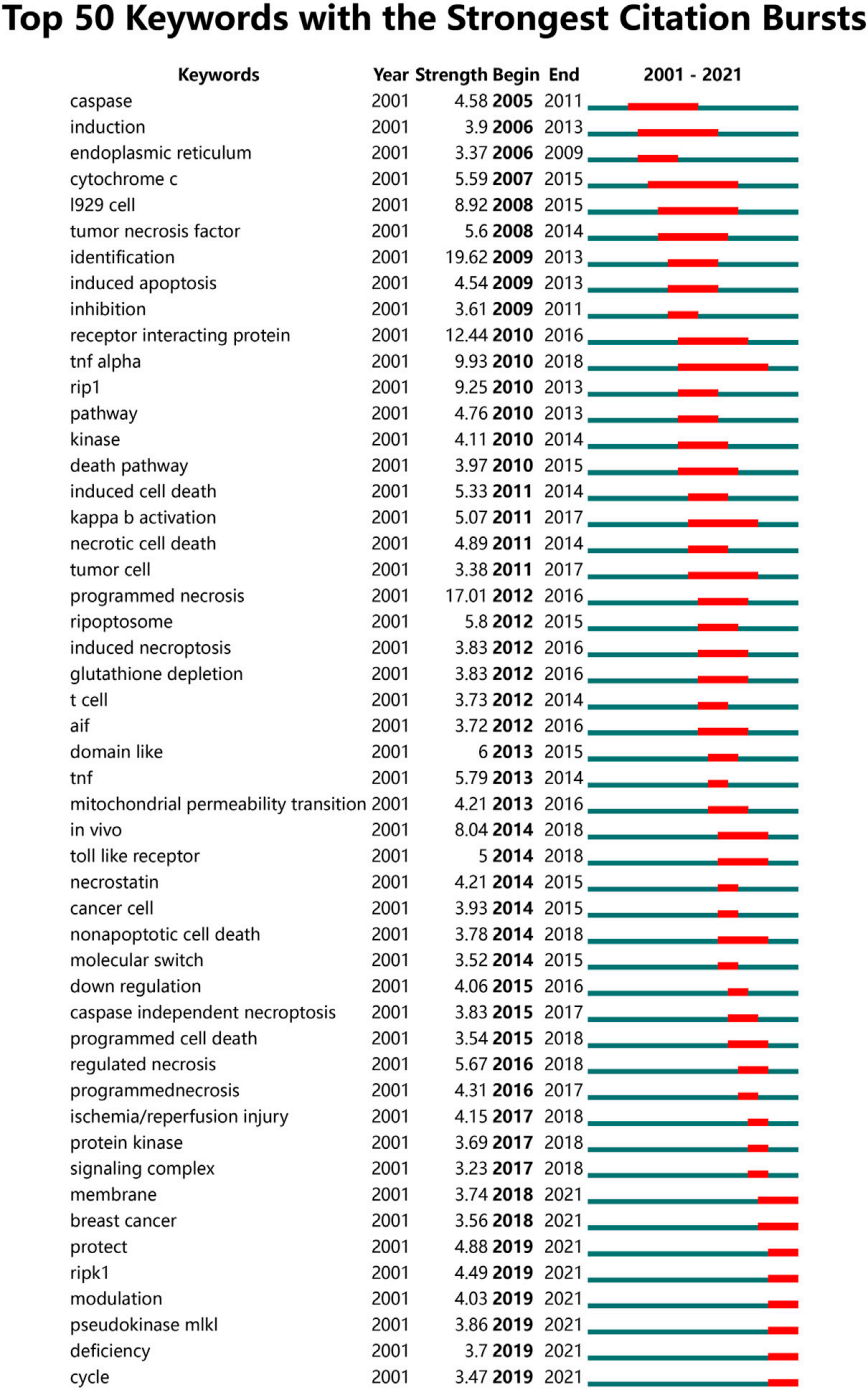
The top 50 burst keywords of necroptosis research.

We identified popular research topics and future research directions in the field of necroptosis by analyzing the extracted keywords. Table 5 shows the top 20 keywords in necroptosis research. Excluding necroptosis (1879), keywords that appeared with the highest frequency in this study were apoptosis (1,481), cell death (812), activation (740), necrosis (643), and inflammation (582). Among these keywords, five appeared more than 500 times, namely apoptosis, cell death, activation, necrosis, and inflammation, suggesting key research topics in this field. Figure 7 shows the 50 most popular keywords with the strongest citation bursts. The top five keywords with the strongest citation bursts included identification, programmed necrosis, receptor-interacting protein, TNFα, and RIP1, suggesting that the immune-inflammatory response pathway is a hotspot. The results also showed that recent studies are increasingly focused on tumor regulation, with these keywords continuing until 2021, including membrane, breast cancer, protection, RIPK1, modulation, pseudokinase MLKL, deficiency, and cycle.

**TABLE 5:** The top 20 keywords related to necroptosis research.

Keyword	Frequency (n)
Necroptosis	1879
Apoptosis	1,481
Cell-death	812
Activation	740
Necrosis	643
Inflammation	582
Programmed necrosis	480
Death	453
Expression	402
Mixed lineage kinase	392
Mechanisms	351
Autophagy	337
Oxidative stress	297
RIP3	282
Kinase	275
NF-κB	275
Phosphorylation	275
Inhibition	267
Protein	256
Cancer	233

## 4 Discussion

In this study, we extracted the literature from public databases and performed a bibliometric analysis to discover and identify popular topics, directions, and future development trends in the field of necroptosis. Our findings show some interesting and thought-provoking points. The annual numbers of academic publications are important indicators of future trends. Before 2013, the number of publications on necroptosis increased relatively slowly each year. However, since 2013, necroptosis-related publications have increased significantly, indicating that necroptosis has attracted significant research interest. There is a relationship between the number of papers published each year and the interest of researchers in this field. The rapid development of necroptosis research over the past decade is remarkable. However, the field of necroptosis research still has many unresolved issues, such as the molecular mechanisms underlying necroptosis and how to reduce the occurrence and development of diseases by inhibiting necroptosis signaling pathways in clinical medicine (Molnar et al., 2019).

Regarding the number of publications in different countries, China occupies a dominant position in the field of necroptosis. Although China publishes the majority of articles, the United States also contributes to many articles. The country corresponding to the distribution of institutions is based on geographical location. Institutions from China (Zhejang University, Fudan University, Shanghai Jiao Tong University, Soochow University, and Sun Yat-sen University) have dominated necroptosis research. Vandenabeele P published the most articles, and Yuan JY was the most influential author with the highest number of citations.

Yuan JY, who previously conducted research at Harvard University, had expert scientific research literacy. Some studies conducted by Yuan et al. discovered necrostatin and its target protein, RIPK1 (Degterev et al., 2005; Ofengeim et al., 2017). They also discovered that activation of RIPK1 promoted necroptosis, while loss of transforming growth factor-β-activated kinase 1 (TAK1) and deficiency of RIPK3 caused necroptosis to lead to apoptosis (Xu et al., 2018). She recently published several high-quality papers on necroptosis in high-impact journals (Xu et al., 2018; Tao et al., 2020; Liang et al., 2021). Wang YY, Zhang J, Xu SW, Young SN, and Czabator PE published more articles recently, especially Wang YY. Among the journals, Cell Death and Disease was the most productive journal in the field of necroptosis research, followed by Cell Death and Differentiation.

Various high-impact studies have focused primarily on basic research related to necroptosis signaling pathways. Reference analysis can identify the underlying mechanism of necroptosis. The most cited and influential reference study demonstrated that the absence of intracellular apoptotic signaling could activate a common non-apoptotic death pathway, called necroptosis (Degterev et al., 2005), which provides the basis for follow-up research on necroptosis. RIP1, RIP3, and MLKL play critical roles in necroptosis, which is executed by oligomerization of MLKL (Park et al., 2021). Sun et al. discovered that MLKL is a key mediator of necrosis signaling downstream of RIP3 kinase through cell experiments (Sun et al., 2012). This study found that RIP3 controls programmed necrosis by initiating the pronecrotic kinase cascade and that this is necessary for the inflammatory response against viral infections (Cho et al., 2009). In addition, a study clarified that RIP3 is the determinant of cellular necrosis in response to the TNF-alpha family of death-inducing cytokines (He et al., 2009). Another study interestingly found that RIP3 did not affect RIP1-mediated apoptosis, but was required for RIP1-mediated necrosis and the enhancement of necrosis by the caspase inhibitor zVAD (Zhang et al., 2009). Based on the analysis and summary above, RIP1, RIP3, and MLKL play an important role in the molecular mechanism underlying necroptosis. These highly cited references provide a certain research basis and direction for future research.

The keywords “necroptosis mechanism” and “molecular” appeared very frequently along with the keywords “RIPK1” and “MLKL” until 2021 as shown in Figure 7. Linear ubiquitination can promote the key protein RIPK1 to inhibit apoptosis and necroptosis, which has important implications for cell survival. RIPK1-knockout mice die due to caspase-8-mediated apoptosis and RIP3-mediated necroptosis (Dillon et al., 2014; Kaiser et al., 2014; Rickard et al., 2014). Furthermore, RIP3 has been reported as a key molecule in necroptosis and has been implicated in the pathogenesis of various cardiac diseases (Karunakaran et al., 2016; Zhu et al., 2018). Two mechanisms have been established to explain how RIPK1 promotes cell survival. TNF-alpha induced protein 3 (TNFAIP3 or A20) and CYLD Lysine 63 Deubiquitinase (CYLD) can deubiquitinate RIPK1, down-regulate RIPK1 and prevent activation of NF-κB signaling (Priem et al., 2019; Lork et al., 202017; Bikker et al., 2017). RIPK1 ubiquitination is essential for TNF activation via the NF-κB signaling pathway (Draber et al., 2015; Roberts et al., 2022). It has been shown that key molecules, pathways, and receptors are important for the NF-κB pathway in necroptosis. Necroptosis is a caspase-independent form of programmed cell death executed by the RIPK1-RIPK3-MLKL signaling cascade (Zhang et al., 2022a). MLKL plays a critical role in necroptosis. RIPK3, and MLKL form the core of the necroptosis machinery, while RIPK1 has dual functions as an important survival factor through its scaffold function and as a mediator of necroptosis through its kinase activity (Pasparakis and Vandenabeele, 2015; Delanghe et al., 2020). Similarly, the keyword “modulation” has also appeared in the past 2 years. Recently, there have been many high-quality reports on the regulation of necroptosis, including those on the interaction between adenosine deaminase acting on RNA 1 (ADAR1) and ZBP1 (de Reuver et al., 2022; Hubbard et al., 2022; Jiao et al., 2022) and the complex protein network of necroptosis (Horne et al., 2022). These results are consistent with our keyword analysis. The molecular mechanism of necroptosis needs to be further studied. In general, the regulation and molecular mechanism of necroptosis is a research hotspot and continues to be of interest in this field.

Furthermore, the keyword “cancer” appeared more than 200 times, and the keyword “breast cancer” appeared until 2021 as shown in Figure 7. Recently, Liao et al. (2022) found indications towards more actionable easy druggable targets and candidate small molecule drugs for potential regulated cell death-related triple negative breast cancer therapies. Some studies have established good prognostic models by analyzing necroptosis-associated genes or miRNAs in breast cancer (Zheng et al., 2022). Many papers have been published on the relationship between necroptosis and cancer (including breast, liver, and prostate cancers) in the last 2 years (Beretta and Zaffaroni, 2022; Li et al., 2022; Wisowski et al., 2022). We conducted a PubMed search for ‘necroptosis and cancer’ and found that the number of articles in 2021 was the highest and continued to increase. A high-quality review published in August 2022 by Peng et al. illustrates the important role of various programmed cell death pathways in cancer, including necroptosis (Peng et al., 2022). Necroptosis follows signal regulation within tumor cells, and after TNF-α binds to TNFR1 on the plasma membrane, downstream protein molecules are recruited to form complex I (Ofengeim and Yuan, 2013). Depending on the microenvironment of cancer cells, complex I activates downstream signaling pathways by regulating RIPK1, which can lead to apoptosis and necroptosis (Chen et al., 2019). Complex I ultimately determines whether cancer cells survive. The choice between apoptosis or necroptosis depends on regulation of the functional conversion of RIPK1. Similarly, a role of ADAR1 and ZBP1 has been reported in cancer (Zhang et al., 2022b). The study demonstrated that ADAR1 inhibits endogenous Z-RNAs and identifies ZBP1-mediated necroptosis as a novel determinant of ADAR1-masked tumor immunogenicity. Therapeutic activation of ZBP1-induced necroptosis brings new perspectives to the field of cancer. This is also consistent with our results, suggesting a close relationship between necroptosis and cancer. These results further prove that the relationship between necroptosis and cancer may be an interesting research direction in the future.

This study had some limitations. First, the included papers published in English were collected from the WOSCC database, which may introduce some inevitable bias. Second, only papers including the terms ‘necroptosis’ or ‘necroptotic’ in the title, abstract, or keywords were retrieved, and those with these terms within the main text were not retrieved for analysis. Moreover, to make the research focus more prominent, we optimized the data analysis results through software parameter adjustments as much as possible, resulting in missing information. Furthermore, keywords with similar meanings can appear in the burst keyword analysis in Figure 7, such as “RIP1”, “RIPK1”, “cancer cell”, “tumor cell”, and “programed necrosis”. In the future, CiteSpace will be updated and made more accurate to overcome this limitation. Further, a previous article reported a similar study on necroptosis (Zhang et al., 2022b), but there are many differences between the two articles. The advantages of our analysis are as follows: our research time span was longer and we included studies from the beginning. Thus, our analysis is more comprehensive and able to reflect the trends and find the early basis and important references in the field of necroptosis (Table 4), which can give direction for follow-up research. In particular, most highly cited papers were published before 2012, which is a clear gap that remained unaddressed in the previous analysis. Furthermore, the emergence of keyword references is more comprehensive and the time span of this emergence is more accurately represented in the present analysis (Figure 7). Finally, regarding analytical methods, Rstudio analysis was used, and thus, some of the results are presented in a clearer and more comprehensible manner in this study, such as those involving the use of bubble charts (Figure 5A).

In conclusion, this is a bibliometric study that provides novel insights into necroptosis using visual analysis software. We found that the relationship between necroptosis and cancer may be a hot topic for future research, and the molecular mechanism of necroptosis requires further study. It also revealed emerging research trends that can help develop a guiding pathway for future research on necroptosis.

## Data Availability

The datasets presented in this study can be found in online repositories. The names of the repository/repositories and accession number(s) can be found in the article/Supplementary Material.
